# A Role for GABA_A_ Receptor β3 Subunits in Mediating Harmaline Tremor Suppression by Alcohol: Implications for Essential Tremor Therapy

**DOI:** 10.5334/tohm.834

**Published:** 2024-04-26

**Authors:** Adrian Handforth, Ram P. Singh, Hovsep P. Kosoyan, Pournima A. Kadam

**Affiliations:** 1 Neurology Service, Veterans Affairs Greater Los Angeles Healthcare System, Los Angeles, California, USA; 2 Research Service, Veterans Affairs Greater Los Angeles Healthcare System, Los Angeles, California, USA

**Keywords:** tremor, cerebellum, alcohol, GABA_A_ receptor, harmaline

## Abstract

**Background:**

Essential tremor patients may find that low alcohol amounts suppress tremor. A candidate mechanism is modulation of α6β3δ extra-synaptic GABA_A_ receptors, that *in vitro* respond to non-intoxicating alcohol levels. We previously found that low-dose alcohol reduces harmaline tremor in wild-type mice, but not in littermates lacking δ or α6 subunits. Here we addressed whether low-dose alcohol requires the β3 subunit for tremor suppression.

**Methods:**

We tested whether low-dose alcohol suppresses tremor in cre-negative mice with intact β3 exon 3 flanked by loxP, and in littermates in which this region was excised by cre expressed under the α6 subunit promotor. Tremor in the harmaline model was measured as a percentage of motion power in the tremor bandwidth divided by overall motion power.

**Results:**

Alcohol, 0.500 and 0.575 g/kg, reduced harmaline tremor compared to vehicle-treated controls in floxed β3 cre- mice, but had no effect on tremor in floxed β3 cre+ littermates that have β3 knocked out. This was not due to potential interference of α6 expression by the insertion of the cre gene into the α6 gene since non-floxed β3 cre+ and cre- littermates exhibited similar tremor suppression by alcohol.

**Discussion:**

As α6β3δ GABA_A_ receptors are sensitive to low-dose alcohol, and cerebellar granule cells express β3 and are the predominant brain site for α6 and δ expression together, our overall findings suggest alcohol acts to suppress tremor by modulating α6β3δ GABA_A_ receptors on these cells. Novel drugs that target this receptor may potentially be effective and well-tolerated for essential tremor.

**Highlights:**

We previously found with the harmaline essential tremor model that GABA_A_ receptors containing α6 and δ subunits mediate tremor suppression by alcohol. We now show that β3 subunits in α6-expressing cells, likely cerebellar granule cells, are also required, indicating that alcohol suppresses tremor by modulating α6β3δ extra-synaptic GABA_A_ receptors.

## Introduction

Observations that low-dose alcohol reduces essential tremor (ET) date back two centuries or more [1,2], yet how it does so has been unexplained. The resolution of this question may lead to effective new therapies. Tremor in ET is reduced by blood alcohol levels of 0.040–0.075 g/dL, below the common driving limit of 0.080 g/dL (17.3 mM) [3,4]. If such blood levels are confined to the arm with brachial artery infusion, tremor suppression does not occur, suggesting that oral doses act within the brain to reduce tremor [5]. Indeed, high-density electroencephalography (EEG) has revealed that tremor reduction by alcohol correlates with alterations of cerebellar activity [6]. The cerebellum displays increased activity in ET as measured with blood flow imaging [7,8]. A tremor-suppressing alcohol dose (blood level = 0.035 g/dL), reduces this hypermetabolism [7], suggesting that in ET cerebellar cortical neurons are hyperactive and that alcohol reduces this hyperactivity.

A candidate mechanism for alcohol’s effect on cerebellum is positive modulation of extra-synaptic GABA_A_ receptors abundantly located on cerebellar granule cells (CGCs). These receptors contain two α and two β subunits, like GABA_A_ synaptic receptors, but incorporate a δ instead of a γ subunit, and exert tonic rather than phasic inhibition. In extra-synaptic receptors δ is associated with α4 subunits throughout most of the brain, but on CGCs a4 subunits are replaced by the closely related α6 subunits, which are highly, and almost exclusively, expressed in CGCs; whereas α4 expression in the cerebellum is very low [9,10]. CGCs from α6 knockout (KO, *α6*^–/–^) mice thus lack GABA-mediated tonic inhibition [11]. Given this location, α6βδ receptor activation or modulation is well positioned to dampen the excitatory CGC drive to Purkinje cells (PCs).

In X*enopus* oocytes expressing recombinant α6β3δ or α4β3δ GABA_A_ receptors, alcohol enhances GABA-mediated tonic currents at levels as low as 3 mM [12], and at levels as low as 10 mM in CGC slices [13,14]. If γ_2_ is substituted for δ in the oocyte recombinant receptors, sensitivity to ethanol is greatly reduced, with threshold effects on GABA currents seen at 100 mM [12]. Thus, δ is required for low-dose alcohol modulation of GABA_A_ receptors. Moreover, modulation of CGC extra-synaptic receptors by alcohol leads to increased GABA release by Golgi neurons via an indirect circuit, so that synaptic GABA_A_ receptors are also activated [15]. These effects of alcohol do not occur in cerebellar slices from *δ*^–/–^ mice [15].

Given the combined clinical evidence and the action of alcohol on α6β3δ CGC GABA_A_ receptors *in vitro* at levels below the driving limit, we have postulated that alcohol suppresses tremor by modulating these receptors [16]. To test this idea we used the mouse harmaline model, in which the brain areas activated during tremor overlap with the tremor circuit revealed by magnetoencephalography in ET [17], and there is considerable pharmacologic overlap, in which numerous drugs exert similar actions on ET and harmaline tremor [18]. We found that low alcohol doses suppress harmaline tremor in wild-type (WT) mice but failed to do in littermates lacking either the δ or the α6 subunit [19]. Moreover, we found that ganaxolone and gaboxadol, which respectively modulate and activate extra-synaptic GABA_A_ receptors, also each suppresses harmaline tremor, but not if either the δ or α6 subunit is lacking [19,20]. The requirement for α6 is also supported by the finding that cerebellar micro-injection of furosemide, an α6 antagonist, blocks alcohol’s suppression of harmaline tremor in mice [21].

In the cerebellum, α6 receptors, both synaptic (α6βγ2) and extra-synaptic (α6βδ), are mainly associated with β2 (51%) and fewer with β3 (21%) [9]. Wallner et al. showed that ethanol enhances GABA currents at levels as low as 3 mM in α4- and α6-β3-δ recombinant receptors in oocytes, but only at 30 mM if the receptors use β2 [12,13]. α6β1δ GABA_A_ receptors are similar to α6β2δ GABA_A_ receptors in their insensitivity to alcohol [22]. In humans, a blood level of 30 mM is highly intoxicating, and in mice the intraperitoneal dose 1.5 g/kg is required to produce this level [23]. Slices from brain areas expressing mainly α4β2δ, such as dentate gyrus or thalamus, display enhanced tonic currents in response to alcohol at 30 mM but not at 20 mM, a response that is absent in slices from δ KO mice [24,25,26,27]. In contrast, slices of CGCs, which express α6β3δ (as well as α6β2δ receptors) [9], respond with enhanced tonic currents at 10 mM [13], findings that are consistent with the oocyte data indicating that β3 confers alcohol sensitivity to δ receptors [12]. Wallner et al. [22] showed that substitution of the normally found tyrosine (Y) at position 66 of β3 by alanine that is normally found in β2 at this position reduces alcohol sensitivity of recombinant α6β3δ GABA_A_ receptors to that of α6β1δ and α6β2δ receptors. In contrast, when serine in position 66 of β1 was replaced by tyrosine, recombinant α6β1δ GABA_A_ receptors now displayed alcohol sensitivity comparable to α6β3δ GABA_A_ receptors [22]. In addition, substitution of the normally found arginine (R) at position 100 of α6 by glutamine further enhances the response to low levels of alcohol in α6β3δ receptors, but not if the receptors use β2 [13], suggesting an interaction between α6 100R and β3 66Y. Wallner et al. concluded that at the α+β– interface in δ-containing receptors these two residues are found at the same interface where they could contribute to a unique alcohol-binding pocket [22].

The β3 subunit thus plays a critical role in mediating a response of GABA_A_ receptors to low-dose alcohol, but does so only in the receptors expressing δ and α4/6. Here we sought to test the hypothesis that mice lacking the β3 subunit will fail to show tremor suppression in response to low-dose alcohol in contrast to littermate controls in which β3 has not been deleted.

## Methods

### Study design

Our goals were to demonstrate that low-dose alcohol can suppress harmaline tremor in WT mice, and to determine whether littermate mice lacking the β3 GABA_A_ receptor subunit fail to respond to this action. An effect of alcohol on tremor was anticipated only in the first post-injection epoch (E1), as alcohol is cleared rapidly in mice [23]. Mice were assigned randomly to dosing groups, and the quantitation was performed by automated software. Animal protocols conformed to the National Institute of Health’s Guide for the Care and Use of Laboratory Animals (Eighth Edition, Washington DC, from the National Research Council, published in 2011), and were approved by the Veterans Affairs Greater Los Angeles Institutional Animal Care and Use Committee. All efforts were made to minimize animal suffering and to reduce the number of animals used.

### Animals

The use of global β3 KO mice to test the hypothesis in the harmaline model is not possible, as these mice are quite abnormal, with high early mortality, cleft palate, seizures, hyperactivity, tremor, and foot clasping [28,29,30]. We therefore employed conditional knockouts, in which part of the β3 gene, flanked by loxP, was deleted only in cells expressing the recombinase cre gene under the control of the GABA_A_ receptor α6 promotor. Mice with the cre gene inserted into exon 8 of the α6 subunit gene on chromosome 11 (B6; D2-Tg(Gabra6-cre) B1Lfr/Mmucd) were obtained from the Mutant Mouse Resource and Research Center at the University of California at Davis (catalog number 015966-UCD). These mice had been backcrossed with C57BL6/J for 5 generations. In our laboratory these mice were backcrossed an additional 5 generations with *δ*^+/+^ (WT) mice, which had been backcrossed to C57BL6/J for 11 generations [19]. This was done to ensure a uniform genetic background among our GABA_A_ receptor subunit colonies, so that results with alcohol should be comparable [19].

Mice with loxP flanking exon 3 of the β3 subunit gene on chromosome 7 (B6; 129- Gabrb3^tm 2.1 Geh^J) were obtained from Jackson Labs (Catalog number: 008310). These mice had been generated on a mixed 129 and B6 background and backcrossed for one generation with C57BL6/J mice. In our laboratory they were backcrossed for 9 generations with *δ*^+/+^ mice.

Once these two lines had each been backcrossed a total of 10 generations, they were interbred to produce a colony that was homozygous for loxPβ3 (referred to as β3^F/F^), and a colony lacking loxPβ3 (WT, referred to as β3^+/+^). Each of these two colonies had cre heterozygotes (referred to as cre+) and cre-negative (cre-) mice. Cre+ mice and cre- mice were interbred to produce littermates for experiments and for further breeding. Genotyping was performed with a polymerase chain reaction (Transnetyx, Memphis, TN). Both sexes were used in experiments.

### Test procedures

To ensure that any reduction in the tremor measure is not due merely to psychomotor impairment, we utilized the straight wire test in β3^F/F^, cre- mice, a sensitive test for psychomotor impairment [31]. In this test, a mouse is suspended by the front paws from a rigid wire, and to pass has to stay on the wire for at least 10 seconds and touch the wire with a hind paw within those 10 seconds, and do so on each test conducted at 10-minute intervals for one hour following alcohol administration. Only doses at which 6/6 mice passed all tests, or lower doses, were utilized in harmaline experiments.

To assess motion power, each mouse was placed on an 8.1-cm diameter mesh on top of a 24.1-cm high cylinder that rested on a 14 × 27.5 cm Convuls-1 Replacement Sensing Platform model 1335-1A (Columbus Instruments, Columbus, OH), fitted in the center with a load sensor, connected to a Grass model P511 AC amplifier (Grass Instruments, West Warwick, RI) with 1 and 70 Hz filter settings. The amplifiers were connected to a desktop computer. The digitally recorded motion power was analyzed using Spike2 software (Cambridge Electronic Design; UK) to perform Fourier transformation of the data into frequency spectra. Up to four mice were tested simultaneously. Data were sampled at 128 Hz. We previously found that harmaline-induced tremor occurs at 9–16 Hz, creating a corresponding motion power peak on digital frequency spectra [32,33]. To control for changes due to activity level, this tremor-associated bandwidth motion power was divided by overall activity motion power to form the measure of analysis, m*otion power percentage* (MPP): (9–16 Hz motion power)/(0.25–32 Hz motion power) × 100, as previously described [33]. The placement of each mouse on an elevated, exposed small platform during motion power accession served to promote sustained alertness with associated tremor.

Mice were acclimated to the platform, then 15 minutes of pre-harmaline baseline motion data collected (referred to as epoch B), then harmaline (Sigma-Aldrich, St. Louis, MO), 20 mg/kg in 4 ml saline/kg injected subcutaneously. Once tremor had developed, within 5 minutes, motion power was again assessed during two successive 15-minute epochs with an intervening 5-minute rest in the home cage (consecutively referred to as H1 and H2 epochs). Ethanol (Thermo Fisher, Canoga Park, CA) was then injected intraperitoneally in doses of 0, 0.40, 0.50, or 0.575 g/kg in saline, 10 ml/kg, as previously described with *δ*^+/+^mice [19]. Motion power accession was re-initiated 10 minutes after injection for four more 15-minute epochs on the elevated platform (E1 to E4), with intervening 5-minute rests.

### Statistical analyses

The motion power percentage (MPP) is defined as the ratio of motion power in the 9–16 Hz bandwidth (numerator) divided by the overall motion power across 0.25 to 32 Hz (denominator).

**Figure 1:**
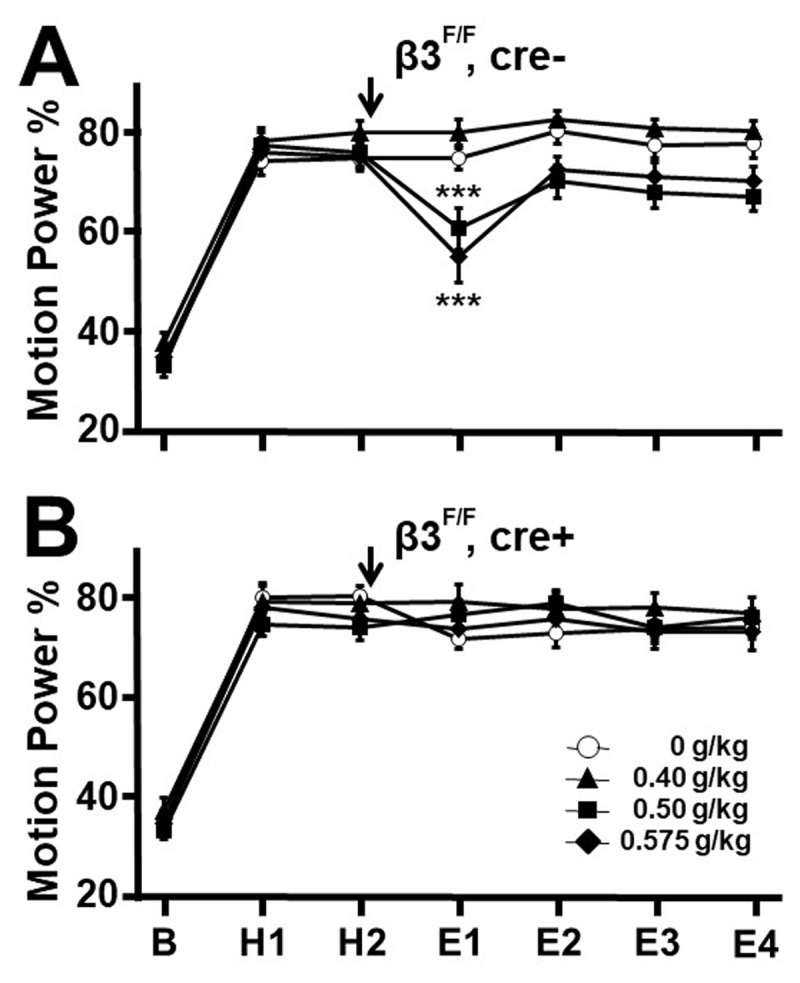
**Effect of cre on harmaline tremor in β3^F/F^mice**. Motion power percentages, calculated as tremor bandwidth motion power (9–16 Hz) divided by overall motion power (0.25–32 Hz) × 100, in groups of mice followed sequentially during 15-minute epochs at baseline (B), pre-treatment harmaline (H1, H2), and after vehicle or alcohol injection (arrow, E1–E4). A. In β3^F/F^, cre- mice, in which loxP flanks exon 3 but excision does not occur, ethanol, 0.575 and 0.50 g/kg but not 0.40 g/kg suppressed tremor during E1 compared to vehicle controls. B. In contrast, in β3^F/F^, cre+ littermates, in which β3 exon 3 is deleted, no dose of alcohol suppressed tremor, indicating the requirement for an intact β3 for the low-dose alcohol anti-tremor response. *p < 0.05, **p < 0.01, ***p < 0.001, ANOVA with Fisher least significant difference criterion.

Mean MPP values, as displayed in Figures 1 and 3, were compared among doses (0, 0.40, 0.50, 0.575 g/kg) and between genotypes using a repeated measure (mixed) analysis of variance (ANOVA) model. A repeated measure model was employed since the same animal is measured repeatedly across 7 time periods (baseline, H1, H2, E1, E2, E3, E4). Residual errors were examined using normal quantile plots (not shown) to confirm that the errors have a normal distribution, as required by this parametric model. Figures 1 and 3 data satisfied the parametric model. The Shapiro-Wilk test for normality also confirmed that the errors followed a normal distribution. The model-based means and pooled standard errors (SEs) were calculated as well as p values for dose comparisons at each genotype-receptor and time.

**Figure 2:**
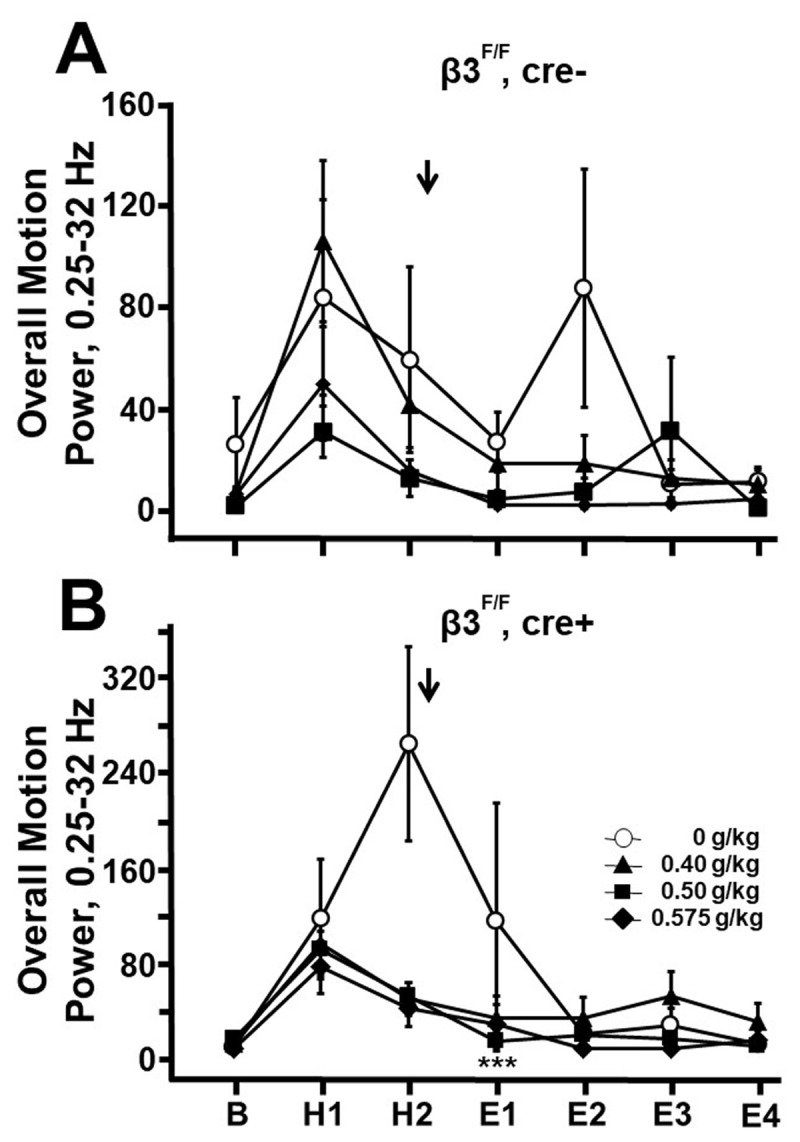
**Effect of cre on overall motion power**. Overall motion power (0.25–32 Hz) values are displayed for the mice whose MPP values are shown in Figure 1. **A.** β3^F/F^, cre-, **B.** β3^F/F^, cre+. Between-genotype comparisons indicate that overall motion power during E1 in β3^F/F^, cre+ mice was greater after 0.50 g/kg than for β3^F/F^, cre- mice, and was comparable for the two genotypes during E1 after 0.575 g/kg alcohol. The failure of β3^F/F^, cre+ mice to show a reduction of MPP during E1 in response to alcohol, as shown in Fig. 1, thus cannot be attributed to abnormally reduced overall motion power, instead the failure of MPP to fall in E1 in this genotype is best explained as a failure to show tremor suppression by alcohol. * *p* < 0.05, ** *p* < 0.01, *** *p* < 0.001, ANOVA with Fisher least significant difference criterion.

Mean overall motion power (0.25–32 Hz) values (not percentages), displayed in Figure 2, were similarly compared using a repeated measure (mixed) ANOVA model. Figure 2 data satisfied the parametric model with the use of the log (base 10) scale. The Shapiro-Wilk test for normality also confirmed that the errors followed a normal distribution. The original scale mean (geometric mean) and its corresponding standard error are reported. Mean differences on the log scale correspond to mean ratios on the original scale.

**Figure 3:**
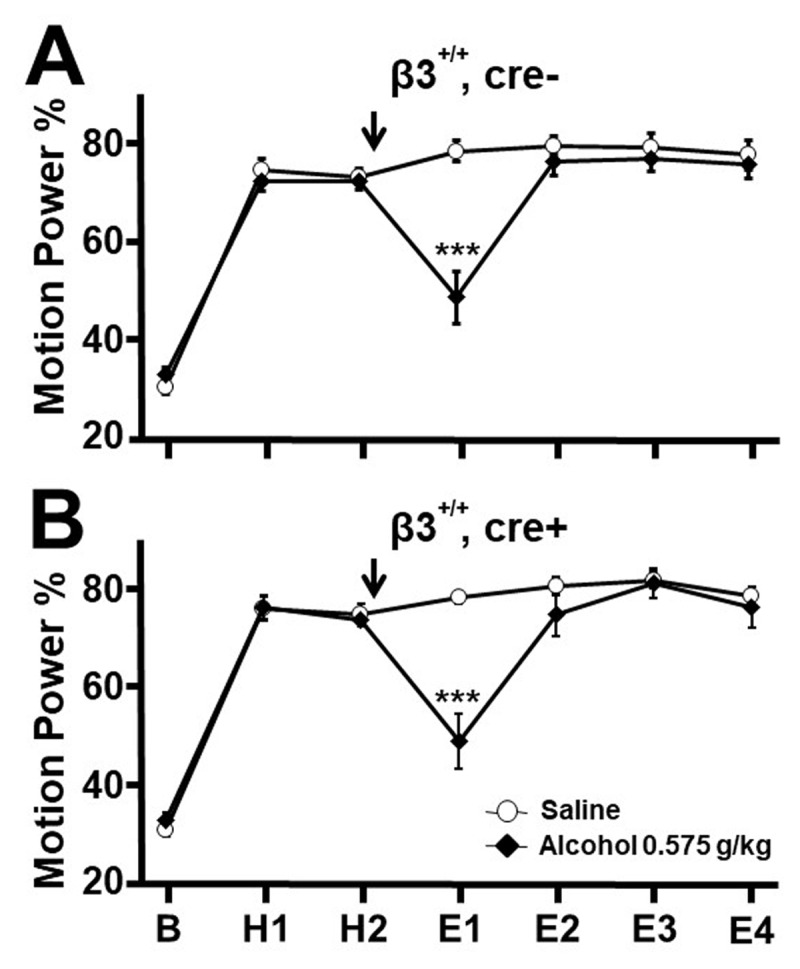
**Effect of cre on harmaline tremor in β3^+/+^ mice**. These mice do not express loxP flanking exon 3 of β3 and so cannot engage in cre-driven recombination. Motion power during baseline (B), pre-treatment harmaline (H1, H2), and after vehicle or 0.575 g/kg ethanol injection (arrow, E1–E4). In **A.** cre-, and **B.** cre+ strains. Ethanol suppressed tremor to a comparable degree in the two genotypes during E1, indicating that the heterozygous insertion of cre into the α6 genome does not by itself interfere with the response to alcohol. * *p* < 0.05, ** *p* < 0.01, *** *p* < 0.001,. ANOVA with Fisher least significant difference criterion.

Mean comparisons under the repeated measure ANOVA models were carried out using the Fisher least significant difference (LSD) criterion (Miller, 1981) [34]. The Fisher LSD allows comparisons among the four dose levels such that the overall chance of a false positive (type I error) is alpha = 0.05 or less. Computations were carried out using R 4.0.5 (R Foundation for Statistical Computing, Vienna, Austria, https://www.R-project.org/).

## Results

### Cre abolishes tremor suppression by alcohol in β3^F/F^ mice

In straight wire testing, 6/6 β3^F/F^, cre- mice passed all tests after alcohol, 0.575 g/kg, thus this and lower doses were used in harmaline experiments. This finding is consistent with previous findings that 0.575 g/kg was passed by 6/6 mice in *α6*^+/+^ and *δ*^+/+^ mice [19], as expected from extensive backcrossing with *δ*^+/+^ so that our subunit colonies shared a common genetic background. The dose of 0.575 g/kg is estimated to produce a blood level of 0.07 g/dL during E1, comparable to blood levels associated with tremor suppression in ET [3,4,23].

In harmaline experiments, the motion power percentage (MPP) that fell by chance within the 9–16 Hz bandwidth was 30–35% during the 15-minute pre-harmaline baseline (B) (Figure 1A, 1B). With harmaline administration, motion power became dominated by tremor, so that the MPP increased to 74–84% during the two 15-minute harmaline pre-treatment epochs (H1, H2). Following injection of saline vehicle or alcohol 0.40, 0.50, or 0.575 g/kg in β3^F/F^, cre- mice, that in the absence of cre have a normally functioning β3 gene [30], (*n* = 12, all groups), tremor was reduced by the 0.50 and 0.575 g/kg doses during post-treatment epoch E1 compared to the vehicle group (Figure 1A, *p* = 0.0007, <0.0001 respectively), but not at 0.40 g/kg. Tremor in the 0.575 and 0.500 g/kg groups recovered during the following epochs, consistent with rapid alcohol clearance [23]. These results are comparable to prior findings that *δ*^+/+^ and *α6*^+/+^ (WT) mice display harmaline tremor suppression in response to alcohol in these doses [19].

Littermate β3^F/F^, cre+ mice in which cre expression occurs under the α6 promotor are conditional KO for exon 3 of the GABA_A_ receptor β3 subunit, so that it is not functioning in cells expressing α6, such as CGCs. These mice displayed normal behavior in the home cage, on handling, and while on the elevated platform, and were indistinguishable from littermate cre- mice. They displayed pre-harmaline baseline and pre-treatment harmaline MPP values comparable to those of cre- mice, indicating no alteration in harmaline tremor response. The normal features of these mice are comparable to the normal behaviors exhibited by floxed β3 mice positive for synapsin 1-cre, in which cerebellar β3 is mostly inactivated [30].

Figure 1B displays motion power in β3^F/F^, cre+ mice receiving vehicle or alcohol 0.40, 0.50, 0.575 g/kg (n = 12, all groups), and shows that, in contrast to β3^F/F^, cre- littermates (Figure 1A), 0.50 and 0.575 g/kg failed to reduce tremor during E1. These findings are interpreted as indicating that the β3 GABA_A_ receptor subunit on α6-expressing cells is required for tremor suppression by low-dose alcohol.

An alternative explanation of the apparent failure of MPP values to fall in β3^F/F^, cre+ mice is that these mice did have alcohol-induced tremor suppression but, owing to a subtle behavioral response to alcohol, overall motion power fell abnormally, so that the resulting MPP values were misleadingly high. In assessing for this possibility, overall motion power (0.25 to 32 Hz) is displayed for these two genotypes in Figure 2. These data are more variable than Figure 1 MPP data as expected given the normalizing effect of the MPP measure. Statistical comparisons required logarithmic conversion to render Figure 2 data parametric. Contrary to the alternative explanation, overall motion power during E1 of β3^F/F^, cre+ mice was greater, not less, than that of β3^F/F^, cre- mice after 0.500 g/kg (0.748 vs –0.337, *p* < 0.001, n = 12, both groups), and was not statistically significantly different on comparing the two genotypes during E1 after 0.575 g/kg (0.267 vs –0.162, *p* = 0.137). These observations support the original interpretation of Figure 1 as indicating that β3^F/F^, cre+ mice fail to show tremor suppression by low-dose alcohol.

### The cre insertion by itself does not interfere with tremor suppression by alcohol

We previously found that whereas *α6*^+/+^ mice respond to 0.500 and 0.575 g/kg alcohol with tremor suppression, their *α6*^–/–^ littermates do not [19]. If the insertion of the cre recombinase gene into part of the α6 genome results in heterozygous expression of the α6 subunit, this could conceivably interfere with suppression of tremor by alcohol, compromising the interpretation of the above results with conditional knockout of β3, which requires the expression of cre. To assess for this possibility, we studied the response of harmaline tremor to alcohol in cre- and cre+ strains that were not floxed (β3^+/+^) so that β3 is intact as no loxP is present to enable exon 3 excision by cre.

In straight wire testing, alcohol 0.575 g/kg did not cause any failures, so that all 6/6 cre- mice and all 6/6 cre+ mice passed, indicating that these genotypes are not more sensitive to the psychomotor impairing effects of alcohol than β3^F/F^, cre- mice tested in the above experiment.

In tremor experiments, saline vehicle or alcohol, 0.575 g/kg, was injected after the second harmaline epoch and then motion power data were accessed starting 10 minutes later. Compared to the vehicle-treated group (n = 12), cre- mice displayed marked tremor reduction during E1 after receiving 0.575 g/kg alcohol (n = 12, *p* < 0.0001, Figure 3A). Cre+ mice displayed MPP values comparable to cre- mice in all epochs, including a marked reduction of tremor in E1 in the alcohol treated group compared to vehicle controls (n = 12, 12, *p* < 0.0001; Figure 3B). This outcome indicates that, by itself, the heterozygous insertion of cre into the α6 genome does not interfere with the ability of low-dose alcohol to suppress tremor.

## Discussion

We found that alcohol in low doses estimated to produce blood levels comparable to those associated with tremor reduction in ET suppressed harmaline tremor in β3^F/F^, cre- mice. In contrast, β3^F/F^ littermates that expressed cre driven by the α6 promotor, so that this recombinase could delete exon 3, inactivating β3, failed to show any tremor suppression to the same doses of alcohol.

We previously found that α6 and δ GABA_A_ receptor subunits are each required for low-dose alcohol’s tremor suppression in the harmaline model [19]. As virtually only CGCs express these subunits together [10,35,36], these cells are very likely the brain site on which alcohol acts to suppress tremor. The validity of the conclusion in the present study that β3 is also required for low dose alcohol’s anti-tremor action requires that the mouse lines we employed demonstrate firstly a high recombination rate within CGCs in response to cre under the α6 promotor, and secondly, that floxed β3 within CGCs is capable of a high rate of allele recombination and inactivation on exposure to an appropriate cre line. Although a limitation of the present study is that we did not confirm the loss of normal β3 in CGCs in our mice, the mouse lines we employed have been characterized and have been shown to satisfy these requirements.

In the study describing the creation and characterization of the D2-Tg(Gabra6-cre) B1Lfr/Mmucd) mouse line, the mice were crossed to a reporter mouse line that expresses *lacZ* upon cre-mediated recombination. They then assessed β-galactosidase expression within brain tissue from adult mice. Marked signal was seen in CGCs, where 92% of cells were positive, and in the cochlear nuclei [37], which express α6 [35,36]. In addition, they noted signal in pre-cerebellar nuclei and in layer 1 of cerebral cortex [37], regions that do not express α6 in adult mice [35,38]. The high recombination rate in CGCs indicates that D2-Tg(Gabra6-cre) B1Lfr/Mmucd) mice are suitable for testing the hypothesis. A tremor effect of low dose alcohol on other nuclei subjected to recombination, but not expressing α6, is not expected to occur. It is conceivable, however, that a loss of β3 in layer 1 of cerebral cortex or in brainstem, if it occurs, could have an indirect effect on behavior that affects the harmaline model by affecting behavior.

Ferguson et al. [30] showed that mice with loxP flanking exon 3 of β3 (β3^F/F^) not exposed to cre exhibit a normal phenotype, suggesting that β3 function is normal in these mice. When they crossed β3^+/F^ mice to an actin-cre transgenic deletor mouse line to recombine the floxed β3 allele and delete exon 3, they found that β3^F/F^, cre+ mice exhibited a severe phenotype resembling that of global β3 knockout mice [29], indicating that widespread deletion of exon 3 by cre in β3^F/F^ mice results in a nonfunctional gene product. They also crossed β3^+/F^ mice to a synapsin I-cre (Syn-cre) transgenic mouse line to produce neuron-specific conditional knockout mice. These mice had higher early-life mortality but were observed to exhibit normal home cage and handling behavior and were fertile, with females showing normal maternal behavior. Western blot with a β3-specific antibody showed marked reduction of β3 in the cerebellum, hippocampus and cerebral cortex, with the cerebellum displaying over 80% reduction [30]. These findings indicate that the 129-Gabrb3^tm 2.1 Geh^J mouse line appears suitable for testing our hypothesis. In addition, this work indicates that marked reduction of β3 in cerebellum is compatible with normal behavior.

Given our prior findings that the α6 subunit is required for tremor suppression by alcohol and by two other drugs that also modulate or activate extra-synaptic GABA_A_ receptors [19,20], it might be conjectured that the failure of β3^F/F^, cre+ mice to manifest tremor suppression with low-dose alcohol is due merely to the heterozygous expression of α6 in cre+ mice. That this is not the case was shown by a control experiment in which cre+ mice lacking loxP displayed just as robust tremor suppression to 0.575 g/kg alcohol as did cre- mice. Yet another possible interpretation of the above finding is that β3^F/F^, cre+ mice metabolize alcohol faster, so that low doses then fail to suppress tremor. That is not likely, as genotypes expressing cre alone or β3^F/F^ alone, bred from the same colony stock, displayed tremor suppression with low-dose alcohol.

The results are consistent with the interpretation that β3 expression on α6 GABA_A_ receptor subunit-expressing cells is required for suppression of tremor by low-dose alcohol. This prediction was made based on studies of recombinant GABA_A_ receptors expressed on oocytes and of slices showing that low alcohol levels modulate α6β3δ receptors, but not α6β2δ or α6βγ receptors [12,13,24,25,26,27].

The GABA_A_ receptor subunit α6 is expressed in the trigeminal ganglion at low levels in association with δ and probably β2/3 [39]. Alcohol is unlikely to be acting here to suppress tremor. Instead, the likely site of alcohol’s action is the cerebellum, where α6 expression is virtually limited to the CGC layer [10]. Insofar as the deletion of β3 in the present experiments by cre under the control of the α6 promotor abolished low-dose alcohol’s anti-tremor action, it may be surmised that alcohol acts on α6β3-containing GABA_A_ receptors on CGCs. Furthermore, given the oocyte and slice data that show that α6β3δ receptors, but not α6β2δ or α6βγ receptors respond to low levels of alcohol, and our previous finding that the δ subunit is required for low-dose alcohol’s anti-tremor action [19], it can be concluded that low-dose alcohol acts on tremor in the cerebellum, where α6β3δ receptors are almost exclusively found. This interpretation is consistent with the observation that intra-cerebellar injection of the α6 antagonist furosemide blocks the anti-tremor effect of ethanol in mice [21], with high-density EEG evidence that alcohol acts on the cerebellum as it reduces tremor in ET subjects [6], and with observations that alcohol reduces cerebellar hypermetabolism in ET [7]. Based on recombinant receptor studies showing a critical role of tyrosine at position 66 in β3 and arginine at position 100 in α6 for conferring high sensitivity to alcohol, Wallner et al. have postulated that these residues in β3 and α6 critically contribute to a unique extracellular binding site for alcohol in α6β3δ GABA_A_ receptors [22].

The local effect of positive modulation of CGC α6β3δ GABA_A_ receptors would be to reduce parallel fiber firing, and thus reduce PC simple spike (SS) firing. How could this suppress tremor? We postulate that the tremorgenic drive to thalamus derives from synchronized deep cerebellar nucleus (DCN) neurons engaged in burst-firing that in turn is driven by excessive PC complex spike (CS) synchrony [16]. It is postulated that the effect of increasing PC SS activity is to enhance PC CS synchrony and promote tremor, whereas reduced PC SS frequency is associated with less PC CS synchrony and amelioration of tremor. The effect of SSs is indirect, via a tri-synaptic pathway. In this circuit PCs that respond with SSs to CGC parallel fibers project GABAergic fibers to DCN neurons that in turn project GABAergic fibers to inferior olivary (IO) neurons that control PC CS synchrony within the same territory affected by parallel fiber input. CSs are spike bursts triggered at IO climbing fiber synapses on PCs [40]. The convergent action of synchronized PC CSs potently inhibits DCN neurons [41,42], provoking hyperpolarization-induced rebound bursting [43] that is transmitted to the thalamus; thus the degree of PC CS synchrony is important for movement amplitude and tremor. Ensembles of PC CSs are synchronized by coupled clusters of projecting IO neurons [44], so that the degree of PC CS synchrony is controlled by the degree of IO coupling. When coupling is increased by local injection of the GABA_A_ receptor antagonist picrotoxin, increased PC CS synchrony and increased movement amplitude ensue [45] and, in some animals, tremor occurs [46]. Similarly, systemic harmaline and intra-olivary serotonin receptor 2a agonists increase IO coupling [47,48,49], increase PC CS synchrony [49,50], and induce tremor [48,51]. In contrast, intra-IO GABA release inhibits coupling, thereby reducing PC CS synchrony [46,52]. The main source of GABA in the IO is the massive GABAergic projection from DCN [53]. These IO-projecting DCN neurons in turn are inhibited by GABA released by PC terminals as PCs engage in SS activity [54,55]. Application of the GABA_A_ receptor agonist muscimol to rat cerebellar cortex reduces PC SS firing, disinhibiting DCN neurons so that they fire more and release more GABA within IO, reducing coupling and therefore PC CS synchrony [56]. As we postulate that excess PC CS synchrony may be associated with tremor [16], such an action would be expected to reduce tremor. In this conceptual framework, CGC activity, as affected by α6β3δ GABA_A_ receptors, controls PC CS synchrony, movement amplitude, and tremor. Enhanced CGC firing, which might occur due to less activation of GABA_A_ receptors or higher afferent drive from brainstem, would increase PC CS synchrony and tremor. Consistent with this notion, ET subjects exhibit high rates of cerebellar metabolism, which may reflect high CGC discharge activity [7,8]. Conversely, low-dose alcohol, by activating CGC α6β3δ GABA_A_ receptors, may exert a muscimol-like action and reduce PC CS synchrony, and thereby ameliorate tremor. In support of this inference, Boecker et al. found that low-dose alcohol reduces cerebellar hypermetabolism in ET patients and moreover increases metabolism in the region of the IO, which they interpreted as due to increased DCN axonal firing [7], comparable to muscimol’s tri-synaptic circuit action in rats [56]. In summary, alcohol’s effect on tremor may be understood as secondary to CGC α6β3δ GABA_A_ receptor-mediated reduction of PC SSs, with downstream reduced PC CS and DCN synchrony via a tri-synaptic circuit.

Ethanol has been shown to exert effects on multiple brain receptors and channels. In many instances, the i*n vitro* or *in vivo* targets are affected at levels above the driving limit of 17.3 mM, such as the AMPA glutamate receptor [57,58], metabotropic GluR4 receptor [59], T-type and L-type calcium channels [60,61], GABA B receptors [62], 5HT3 receptors [63], adenosine regulation [64], and GIRK2 (G-protein inwardly rectifying potassium current) [65]. A few targets have been reported to be affected by non-intoxicating alcohol levels, including inhibition of NMDA receptors [66], metabotropic GluR1 [67], large-conductance potassium (BK) channels [68], and α6 subunit-containing nicotinic receptors [69]. It may be noted, however, that in our experiments with mice administered alcohol 0.50 or 0.575 g/kg, tremor suppression occurred in wild-type mice but not in littermates lacking the α6, the β3, or δ GABA_A_ receptor subunits, indicating that any alcohol effect on alternative targets was not sufficient to affect tremor at thee doses. Several of the targets listed above have anti-tremor potential as in the case of NMDA receptor antagonists, AMPA receptor antagonists, and GABA B receptor agonists [18], but such targets have potential disadvantages associated with widespread expression in the brain, whereas α6β3δ GABA_A_ receptors are virtually confined to a focus of efficacy, the CGC.

In conclusion, our results, in combination with our earlier findings [19], suggest that low-dose alcohol suppresses tremor by modulating α6β3δ extra-synaptic GABA_A_ receptors on CGCs. The postulated anti-tremor mechanism is a reduction of PC CS synchrony, so that excessive DCN and hence thalamic synchrony is lessened. The results do not imply that α6β3δ is the only viable target among GABA_A_ receptors for novel anti-tremor drugs. The localization of α6β2δ and α6β2/3γ2 GABA_A_ receptors on CGCs also render these as attractive therapeutic targets [70].
